# Supplementary material for: Decreased Expression of CD314 by NK Cells Correlates with Their Ability to Respond by Producing IFN-γ after BCG Moscow Vaccination and Is Associated with Distinct Early Immune Responses
Source: Vaccines (Basel). 2023 Jul 28;11(8):1297. doi: 10.3390/vaccines11081297 (PMC10458680; doi:10.3390/vaccines11081297)
Supplement: Supplementary file 1 [file vaccines-11-01297-s001.zip › vaccines-2516387-supplementary.pdf]

Supplementary Material

Decreased Expression of CD314 by NK Cells Correlates with Their Ability to Respond by Producing IFN-γ after BCG Moscow Vaccination and Is Associated with Distinct Early Immune Responses

Adeliane Castro da Costa [1,2], Lília Cristina de Souza Barbosa [2], André Kipnis [2] and Ana Paula Junqueira-Kipnis [2,*]

[1] Campus Goiânia, Goiás Estácio de Sá University, Goiânia, ZC 74063-010, Brazil; adeliane.castrodacosta@gmail.com
[2] Institute of Tropical Pathology and Public Health, Department of Biosciences and Technology, Federal University of Goiás, Goiânia, ZC 74605-050, Brazil; lcristinasb19@ufg.br (L.C.d.S.B.); akipnis@ufg.br (A.K.)
* Correspondence: ana_kipnis@ufg.br

1 Supplementary Tables and Figures

1.1 Supplementary Tables

Table S1. Fluorescent monoclonal antibodies and clones used in the study.

Fluorochrome	Marker	Catalog #	Clone
FITC	CD16	11-0168-42	eBioCB16 (CB16)
FITC	CD4	11-0047-42	SK3 (SK-3)
PE	CD49d	12-0499-42	9F10
PE	IFN-γ	12-7319-42	4S.B3
PE	TNF-α	12-7349-82	MAb11
PE	CD8a	12-0087-42	SK1
PercP	CD63	MA110269	MEM-259
PerCP-eFluor™ 710	CD314 (NKG2D)	46-5878-42	1D11
PE-Cyanine7	CD15	25-0159-42	HI98
PE-Cyanine7	TCR V alpha 24 J alpha 18	25-5806-42	6B11
PE-Cyanine7	IL-22	25-7229-42	22URTI
eFluor® 660	CD57	50-0577-42	TB01 (TBO1)
APC	CD27	17-0279-42	O323
APC	CD66	17-0668-42	CD66a-B1.1
APC	LAP (Latency Associated Peptide)	17-9829-42	FNLAP
APC	IL-2	17-7029-41	MQ1-17H12
Alexa Fluor 700	CD3	56-0038-42	UCHT1
Alexa Fluor 700	ARGINASE	56-3697-82	A1exF5
Alexa Fluor 700	CD14	56-0149-42	61D3
Alexa Fluor 700	IFN-γ	56-7319-42	4S.B3
APC-eFluor® 780	CD56 (NCAM)	47-0452-82	RA3-6B2
APC-eFluor® 780	CD123	47-1239-42	6H6
APC-eFluor® 780	CD16	47-0168-42	eBioCB16 (CB16)
APC-eFluor® 780	IL-17A	47-7179-42	eBio64DEC17

1.2 Supplementary Figures

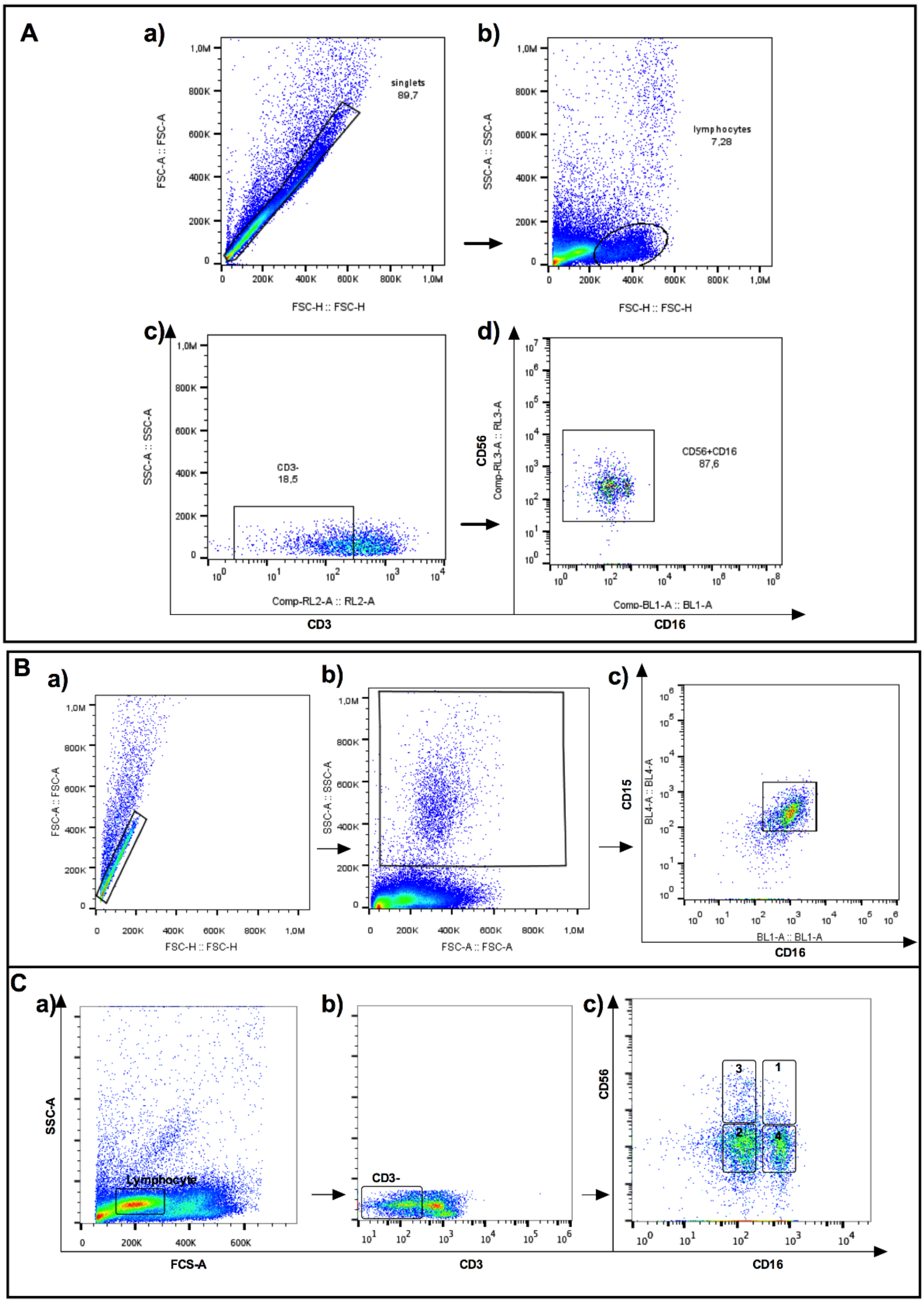

Figure S1. Gating strategies. All cells were gated to exclude doublets using FCS-A and FCS-H parameters. **A)** To evaluate NK cells, at least 30,000 events were acquired and analysed. After this, based on granularity and size,

lymphocytes were gated **(b)**. Then CD3$^-$ cells were selected **(c)**, and the CD56$^+$ cells were considered NK cells **(d)**. **(B)** For neutrophils, 200,000 events were acquired, and singlets **(a)** were analyzed for size and granularity (b, FSC and SSC) and the cells were gated using CD15 and CD16 cells markers **and** neutrophils were considered CD15 $^+$CD16$^+$ **(c)**. **C) Gating strategy for NK subpopulations**: Lymphocyte singlets (a) were gated and CD3 negative lymphocytes (b) were evaluated for the expression of CD56 and CD16 (c). NK subpopulations were defined based on the expression of CD16 and/or CD56 as: Gate 1: CD56^{bright}CD16$^+$, Gate 2: CD56^{dim}CD16$^-$, Gate 3: CD56^{bright}CD16$^-$, and Gate 4: CD56^{dim}CD16$^+$. The cells were acquired using an Attune™ NxT flow cytometer. The data were evaluated using FlowJo software version 10.1.

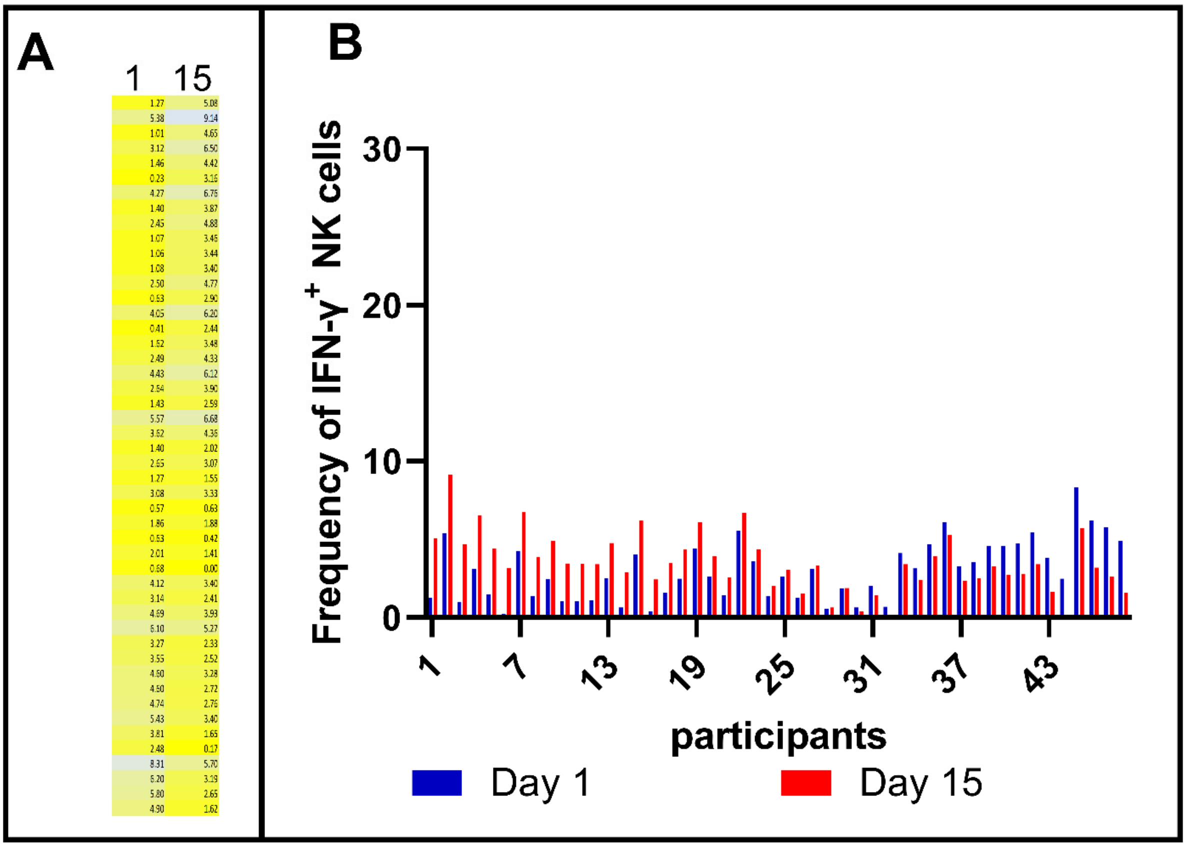

Figure S2. IFN-γ+ NK cells of individuals not vaccinated with BCG Moscow on days 1 and 15 were classified according to their IFN-γ+ percentage variation. In A, the IFN-γ+ NK cells were classified pairwise according to the response magnitude. In B, the results from the first column are shown as blue bars and correspond to day 1 for each participant. The red bar represents paired IFN-γ+ NK cell MFI 15 days postvaccination. The Y-axis is on the same scale as Figure 1 to allow for responsecomparisons.

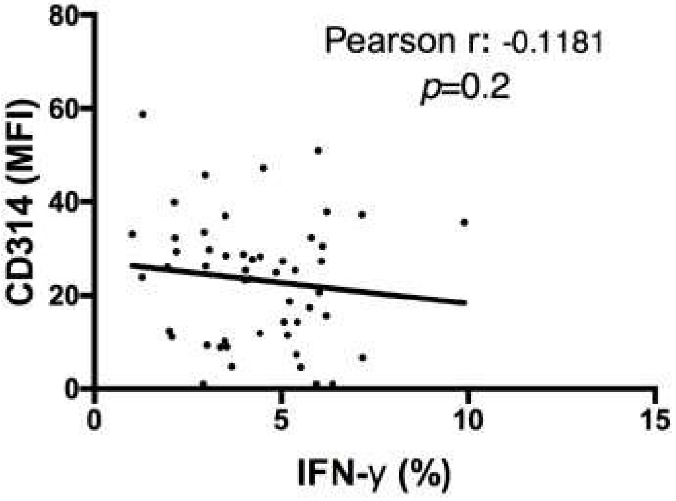

Figure S3: Correlation between the NK cell MFI of CD314 at baseline and the percentage of CD4-IFN-γ^+ T-cells 15 days post-BCG vaccination. r: The Pearson test correlation value and p value are shown inside the graph.
